# Impact of COVID-19 pandemic on the professional intention of medical and related students

**DOI:** 10.1186/s12909-021-02922-2

**Published:** 2021-09-09

**Authors:** Zheng Gong, Wen Li, Huimin Bu, Mingyu He, Hongjian Hou, Tongtong Ma, Xide Hu, Lu Fu, Joseph Adu-Amankwaah, Hong Sun

**Affiliations:** 1 Department of Physiology, School of Basic Medicine, Xuzhou Medical University, No.209 of Tongshan Road, Xuzhou, 221004 Jiangsu China; 2 School of Public Affairs & Governance, Silliman University, Dumaguete, Philippines; 3 School of International Education, Xuzhou Medical University, Xuzhou, Jiangsu China; 4 College of Biology and Food, Shangqiu Normal University, Shangqiu, Henan China; 5 Department of Human Physiology and Pathophysiology, Kangda College of Nanjing Medical University, Lianyungang, Jiangsu China

**Keywords:** COVID-19, Medical, Health, Students, Major, Workplace, Career intentions

## Abstract

**Background:**

The outbreak of COVID-19 has led to increased workload and infection risks among medical staff. This situation may influence current medical and health-related students’ decision on the choices of their future careers. Hence, this study investigated the impact of COVID-19 on their future career intentions.

**Methods:**

This is a cross-sectional observational study that included medical and health-related students from three universities between October 2020 and January 2021. The study questionnaire was divided into two main sections: Section 1, which comprised students’ basic information. And section 2 focused mainly on the impact of COVID-19 pandemic on students’ professional intentions. The chi-squared χ^2^ test was used to compare the responses before and after the pandemic outbreak among Chinese and non-Chinese students.

**Results:**

In overall, 1253 students completed the questionnaires. The responses showed that the number of students who preferred clinical medicine, public health, pharmacy and oral medicine increased significantly after the pandemic outbreak. In contrast, the number of students who chose nursing and medical technology decreased significantly. The change mainly occurred in Chinese students, predominantly females. Half of students (50.35%) were more willing to engage in medical and health work after completing their current program. Also, 36.39% of students felt that knowledge was too limited in the pandemic’s face and would like to continue studying after graduation to gain more knowledge. Due to the pandemic, 34.18% of students would like a future workplace near their hometown, and 19.63% preferred to work in urban areas.

**Conclusion:**

The COVID-19 outbreak impacted current medical and health-related students’ career planning on their future workplaces and employment time choices. Additionally, the pandemic influenced the intention of Chinese students in choosing their future careers. This study provided the basis for the policymaking, specialty setting of colleges and supplied the medical health department’s talent reserve information.

## Background

In 2020, the global COVID-19 brought great changes to all walks of life [1]. The future world will be rewritten by the pandemic, just like the Spanish influenza, influenza, smallpox, cholera, atypical pneumonia and other pandemics that the world suffered in the past 100 years or so, which had a huge impact on society [2–5]. In history, large-scale epidemics forced mankind to break away from the past and reopen time. The same is true of COVID-19.

For college students, education is to prepare for the next stage of life. Therefore, they will certainly focus on the changing external environment. COVID-19 keeps them on the verge of change. In the face of the pandemic, doctors, nurses, public health workers and other health related workers are the hardest, most tired and most risky. They are fighting on the front line, not only working overtime, but also risking infection [6, 7]. Seeing this situation, will medical and health-related students waver in their major choice? In this study, questionnaire survey was conducted to determine the impact of the COVID-19 pandemic situation on professional intents of medical and health related students.

## Methods

### Formation of questionnaire

Seven Chinese and three non-Chinese medical students were interviewed to discuss the factors that may influence their future careers choices due to the COVID-19 pandemic. A questionnaire was formed based on the responses from the interviews, which consisted of two sections. Section one comprised the basic information of students, such as the age, gender and nationality. And section two focused mainly on the impact of the COVID-19 pandemic on students’ future professional intentions. This section included: Students’ current majors; and the majors the students preferred to study due to the influence of COVID-19, their preferred future workplaces and working time. The methods used in this study were carried out in accordance with relevant guidelines and regulations. This study was approved by the Ethics Committee of Xuzhou Medical University (See attachment).

### Data collection

Teachers distributed the electronic questionnaire to students of three universities, namely, Xuzhou Medical University, Shangqiu Normal University, Kangda College of Nanjing Medical University. A total of 1260 questionnaires were collected. Seven uncompleted questionnaires were excluded, and 1253 valid questionnaires were obtained. Out of the 1253 were Chinese and non-Chinese students from seven different majors, including medicine, nursing, public health, medical technology, pharmacy, stomatology and basic medical science (including biology). Informed consent was obtained from all participants or, if participants were under 18, from a parent and/or legal guardian.

### Statistical analysis

Chi-square (and Fisher’s exact test) and GraphPad Prism 7.00 were used to analyze the data obtained. Mean differences were compared with statistical significance set at *p* < 0.05. Results were represented as tables and graphs.

## Result

### The general profile of students

A total of 1253 medical and health-related students from 25 countries in Asia, Africa and Oceania participated in the questionnaire survey. Out of the total, 842 were females, and 411 were males. Their ages ranged from 18 to 27 years, with an average age of 20 ± 2 years. Of the total students, 1128 were Chinese, consisting of 770 females and 358 males; and 125 were non-Chinese and consisted of 72 females and 53 males, mainly from India, Nepal and other countries in Africa and South Asia (Table 1).

**Table 1:**
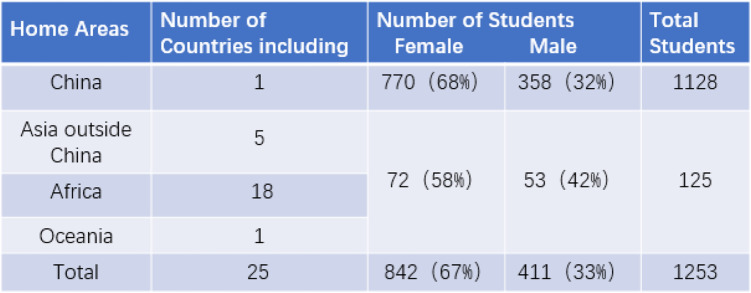
Information of students participating in the questionnaire

### The impact of COVID-19 pandemic on continuing study of medical and health related major

**Fig. 1:**
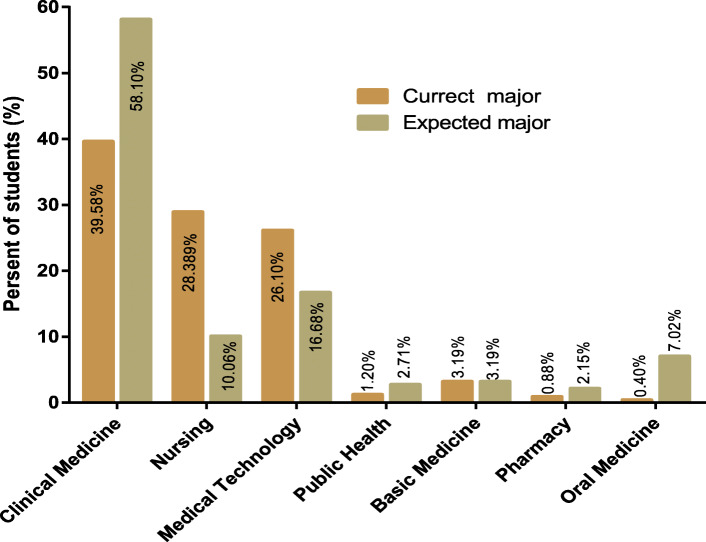
An overview of students’ major selection before and after the pandemic. A total of 1253 students participated in the survey

Compared with before the outbreak of the COVID-19 pandemic, the number of students who preferred clinical medicine, public health, pharmacy and oral medicine increased significantly after the pandemic outbreak. In contrast, the number of students who chose nursing and medical technology decreased significantly. However, the number of students who preferred basic medicine remained unchanged. The percentage of students regarding their major choices before and after the epidemic was shown in Fig. 1.

**Fig. 2:**
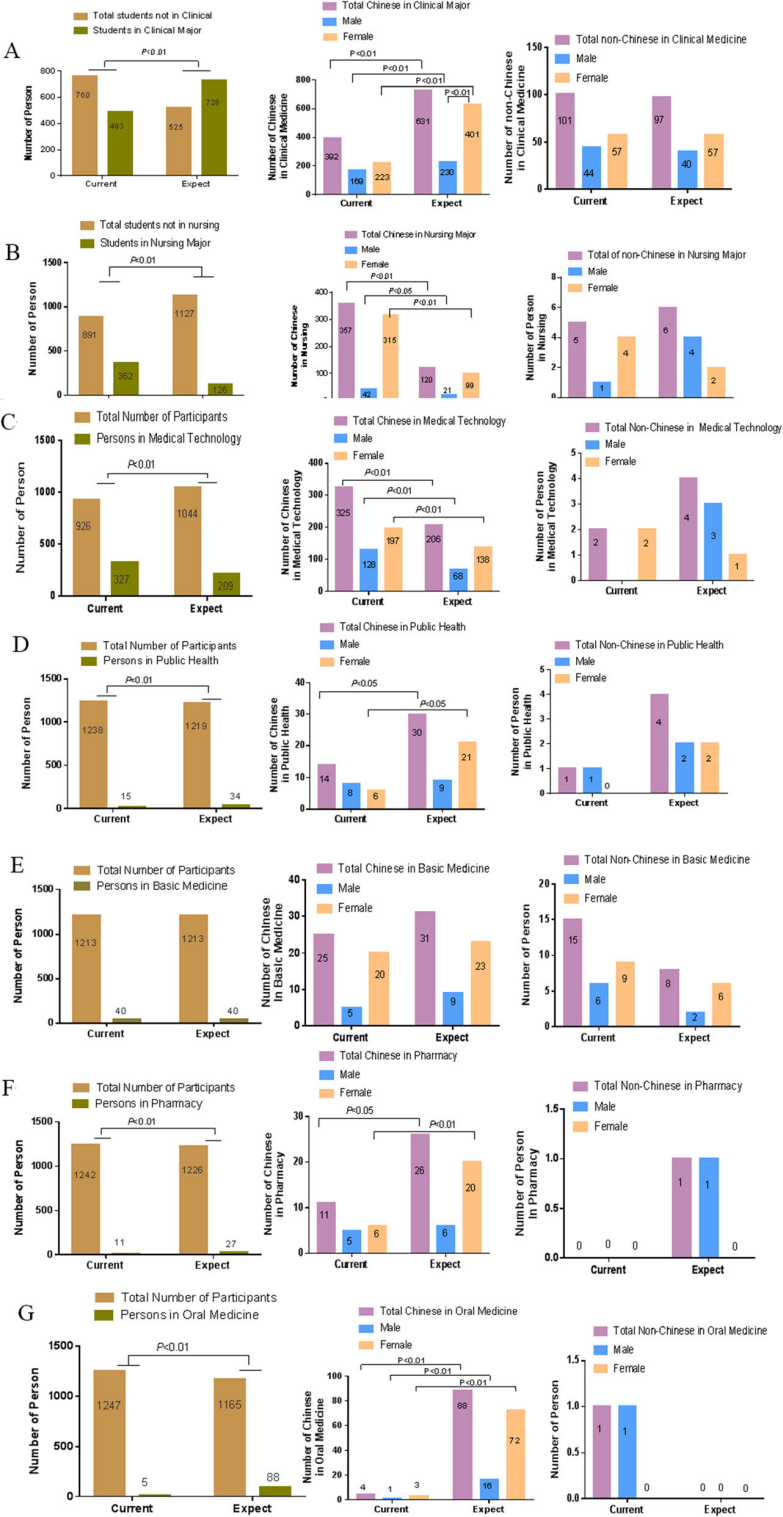
Impact of COVID-19 pandemic on students’ choice of major

The change in major choices due to the COVID-19 pandemic mainly occurred in Chinese students but not in non-Chinese students (Fig. 2).

Among the Chinese, the number of students choosing clinical medicine and oral medicine increased significantly after the pandemic (*P* < 0.01) (Fig. 2A, G). On the flip side, the number of students who preferred nursing and medical technology decreased significantly (*P* < 0.01) (Fig. 2B, C). Also, the number of Chinese female students choosing public health increased (*P* < 0.05) compared to males (Fig. 2D). Although there was no statistical difference, the number of non-Chinese students choosing basic medicine had an increasing trend (Fig. 2E).

Comparison of the number of students who had chosen major before pandemic with those who wanted to choose after pandemic in Clinic Medicine(A), Nursing(B), Medical Technology(C), Public Health(D), Basic Medicine(E), Pharmacy(F), and Oral Medicine(G), respectively. A total of 1253 students participated in the survey, in which 1128 students were Chinese, including 770 females and 358 males, and 125 students were non-Chinese, including72 females and 53 males. Chi-square (and Fisher’s exact test) were conducted to test for differences before and after pandemic. Statistical significance was set at *p* < 0.05.

### Influence of COVID-19 pandemic on the select intents after completing current program

The pandemic influenced the plans of students after the completion of their current program. Interestingly, half of the students (50.35%) were more willing to engage in medical and health work, notwithstanding the fact the epidemic increased the workload and risk of medical staff (Table 2). However, only a tiny minority of students (1.51%) were terrified to practice as medical and health workers because of the pandemic and planned to change their future profession if possible. These changes were evident among Chinese and non-Chinese male students (*P* < 0.05). Also, 36.39% of the students felt that knowledge was too limited in the pandemic face and would like to continue their studies after graduation. In contrast, 147 of students (11.73%) planned to work immediately after graduation due to financial difficulties of their families caused by the pandemic.

**Table 2:** Impact of epidemic situation on the next plan after completing current program

Items	Total	Number of students Chinese Total Female Male			Number of students non-Chinese Total Female Male		
1. The pandemic has changed the financial situation of my family, so I intend to work after completing current program.	147^**^	109^**^	67^**^	42^**^	38^# &&^	24	14
2. The pandemic has made me more aware of the significance of medical and health work, and I am more willing to engage in medical and health work. I intend to work after completing current program.	631^**^	566^**^	398^**^	168	65^**^	42	23
3. The epidemic made me realize that my knowledge is limited. If possible, I want to continue to study after graduation	456^**^	434^**^	292^**^	142	22^&&^	6^@^	16
4. The pandemic made me terrified to practice in medical and health work, so I plan to change my profession, if possible.	19	19	13	6^**^			
Total	1253	1128	770	358	125	72	53

Chi-square (and Fisher’s exact test) were conducted to test for differences. Statistical significance was set at *P* < 0.05. ^**^P < 0.01 compared with the choice of other items in same column; ^#^*P* < 0.05 compared with the choice of items 3; ^&&^ *P* < 0.01 compared with corresponding total Chinese; ^@^*P* < 0.05 compared with corresponding non-Chinese male

### The impact of COVID-19 pandemic on the choice of working place

Although, the pandemic did not affect the choice of working places of 45.17% students, it did influence majority of them (Table 3). Among the majority, 246 students (19.63%) thought the closer of working place near hometown, the better, while 183 students (14.55%) preferred to go back home directly. However, 246 students (19.63%) preferred to work in big cities. Only a few students (0.95%) have no plans for their future workplace.

**Table 3:** The effect of pandemic on the choice of working place

Items	Total	Number of students Chinese Total Female Male			Number of students non-Chinese Total Female Male		
1. Affected by the COVID-19 epidemic, I prefer to go back to my hometown to work.	183^**^	162^**^	101^**^	61	21	11	10
2. Affected by the COVID-19 epidemic, I think the closer of my working place to my hometown, the better.	246	221	157	64	25	14	11
3. Affected by the pandemic, I prefer big city, even if it’s far away from hometown.	246	226	140^&^	86	20	13	7
4. The COVID-19 epidemic will not affect my selection of work location.	566^**^	507^**^	366^&**^	141^@^	59	34	25
5. I’m not sure now	12^**^	12^**^	6^**^	6^**^			
Total	1253	1128	770	358	125	72	53

Chi-square (and Fisher’s exact test) were conducted to test for differences. Statistical significance was set at *P* < 0.05. ^**^P < 0.01 compared with the choice of other items in same column; ^&^*P* < 0.5 compared with corresponding Chinese male; ^@^ *P* < 0.05 compared with the choice of items 1, 2, 3

Among the Chinese students, females were less affected by the pandemic on choosing their workplaces compared to males, with fever students preferring big cities. However, there was no significant difference in workplace choice between non-Chinese males and females, and between Chinese and non-Chinese.

## Discussion

Our study found that the COVID-19 pandemic had a great impact on the future career planning of medical students and health-related students. Compared with before the pandemic, the number of students willing to choose for all majors except basic medicine has changed significantly. In the choice of future workplace, more than half of the students were affected by the pandemic. The pandemic also had a huge impact on the choice of either continuing to study or practicing as a medical and health worker after graduation.

The outbreak of the pandemic has made a lot of countries feel the obvious lack of medical staff. Simultaneously, people are more aware of the workload and risks involved in medical and health-related work [8]. Interestingly, the findings of this survey revealed that more students would like to choose the profession of clinical medicine and public health after the outbreak of the pandemic, which was mainly seen among Chinese students, especially females. In recent years, the shortage and turnover of medical staff have been reported sometimes, with some medical staffs given up their medical work due to stress problems, life-work unbalance and medical disputes [9–11]. After the outbreak of the pandemic in China, the whole society highly appraised the contribution of medical staffs in fighting against the pandemic [12]. They are more respected, and their social status in peoples’ minds has been greatly improved, enhancing their sense of pride and self-confidence. We believe that this is at least one reason why more medical and health-related students are willing to engage in medical and public health work in the near future.

Additionally, this study showed that the number of students who preferred nursing and medical technology were significantly reduced after the pandemic outbreak, which mainly occurred among Chinese students, predominantly females. Usually, nurses and medical technicians’ hard works are not visible, making their efforts less appreciated by the public. This may eventually lower their self-confidence and social status compared with that of doctors and public health professionals. Therefore, creating the awareness of the significant roles played by nurses and medical technicians will make the public realize that their work is an indispensable part of the medical process. This will go a long way to enhance the respect and social status of these professionals. Additionally, providing them with a good life and improving their working conditions is key in attracting more students in choosing these professions in the near future.

Basic medical science is the bedrock of clinical medicine and is the premise for medical and public health development. The number of students who preferred the major of basic medicine remained the same, indicating that the enthusiasm of students for basic medicine did not change despite the pandemic.

In this study, we found that non-Chinese students’ major choices were not significantly affected by the pandemic. This was also reflected in the interviews with students. It is crucial for non-Chinese students to stick to their plans of studying medicine and related majors based on their decisions made before entering China. Thus, their families and relatives agreed to study their current major after considering the advantages and disadvantages of all aspects. Therefore, ideologically, it is difficult for them to change their majors. The professional stability of this group of students is better.

However, among the Chinese students, females were more affected by the pandemic in their choices of major. This contradicts traditional Chinese feminism, which is being afraid of change and unwilling to take risks. There may be two reasons for this: firstly, quite a lot of Chinese medical students’ majors are chosen based on the decision of parents, social orientation or career income, rather than their own choice after the college entrance examination. Secondly, as modern intellectuals, female college students’ traditional role expectation conflicts with modern equal gender culture, and they also pay more attention to the realization of self-value. These make it easy for them to change their majors when faced with big challenges, such as the COVID-19 pandemic. Further detailed studies to investigate factors affecting professional stability of Chinese females are needed.

## Conclusion

In conclusion, the COVID-19 epidemic influenced the career planning of Chinese and non-Chinese medical and health-related students in choosing their majors, workplaces and employment time. Medical and health-related students are the successors of medical and health undertakings in the near future. The particularity of the medical and health profession determines the importance of medical and health-related students’ professional identity. Therefore, the state and university (college) should pay more attention in the training of medical and health professionals, enhancing their professional identity and sense of mission, and also ensuring the reserve of medical and health-related personnel.

This study provides the basis for administrative departments’ policy making, professional setting and enrollment of medical colleges, and the talent reserve information for the next employment plan of the medical and public health department.

## Data Availability

The datasets used and/or analysed during the current study are available from the corresponding author on reasonable request.
